# Development of a Hydrogen Peroxide Sensor Based on Screen-Printed Electrodes Modified with Inkjet-Printed Prussian Blue Nanoparticles

**DOI:** 10.3390/s140814222

**Published:** 2014-08-04

**Authors:** Stefano Cinti, Fabiana Arduini, Danila Moscone, Giuseppe Palleschi, Anthony J. Killard

**Affiliations:** 1 Dipartimento di Scienze e Tecnologie Chimiche, Università di Roma Tor Vergata, Via della Ricerca Scientifica 1, Rome 00133, Italy; E-Mails: Stefano.Cinti@uniroma2.it (S.C.); Fabiana.Arduini@uniroma2.it (F.A.); Danila.Moscone@uniroma2.it (D.M.); Giuseppe.Palleschi@uniroma2.it (G.P.); 2 Department of Biological, Biomedical and Analytical Sciences, Faculty of Health and Applied Sciences, University of the West of England, Coldharbour Lane, Bristol BS16 1QY, UK

**Keywords:** hydrogen peroxide, Prussian blue nanoparticles, screen-printed electrode, inkjet printing

## Abstract

A sensor for the simple and sensitive measurement of hydrogen peroxide has been developed which is based on screen printed electrodes (SPEs) modified with Prussian blue nanoparticles (PBNPs) deposited using piezoelectric inkjet printing. PBNP-modified SPEs were characterized using physical and electrochemical techniques to optimize the PBNP layer thickness and electroanalytical conditions for optimum measurement of hydrogen peroxide. Sensor optimization resulted in a limit of detection of 2 × 10^−7^ M, a linear range from 0 to 4.5 mM and a sensitivity of 762 μA·mM^−1^·cm^−2^ which was achieved using 20 layers of printed PBNPs. Sensors also demonstrated excellent reproducibility (<5% rsd).

## 1. Introduction

The effective measurement of hydrogen peroxide remains a critical analytical goal due to its widespread use in various fields such as food processing, the textile industry, pulp and paper bleaching, pharmaceutical research, clinical chemistry, antiseptic and disinfecting agents [1–3]. H_2_O_2_ is also involved in several biological events and intracellular pathways and is the by-product of enzymatic processes such as those involving, for example, glucose oxidase, cholesterol oxidase and alcohol oxidase. H_2_O_2_ is also a substrate for the enzyme horseradish peroxidase [4]. Reliable, accurate, sensitive, rapid, and cost-effective determinations of H_2_O_2_ continue to be widely investigated.

Several analytical techniques including spectrophotometry [5], chemiluminescence [6] and fluorescence [7] have been employed in the determination of H_2_O_2_. However, most of them exhibit their own technical drawbacks such as being time consuming, complicated or requiring expensive instrumentation. The area of printed electrochemical sensors has proven successful for the detection of many analytes owing to their sensitivity, selectivity, fast response, ease of use and cost-effectiveness. Together with progress in the field of nanomaterials, sensors are an excellent choice in the realization of the accurate and sensitive detection of H_2_O_2_. Significant effort has been expended on the design of novel H_2_O_2_ sensing techniques and the improvement of their analytical performances.

A wide range of materials such as redox proteins, transition metals and redox polymers have been employed to perform electrocatalytic H_2_O_2_ detection [8–11]. Ferric hexacyanoferrate or Prussian blue (PB) has been referred to as an “artificial peroxidase” [12]. It is electrochemically reduced to form Prussian white (PW), which is capable of catalyzing the reduction of hydrogen peroxide at low potentials (around 0 V *vs.* Ag/AgCl), which is an excellent characteristic as it allows it to work in the presence of a wide range of interferences. There are several methods in the literature for producing PB. Almost all of the procedures adopted for deposition on electrode surfaces are based on electrochemical techniques [13] or *in situ* chemical deposition [14]. However, PB itself can also be fabricated into different nanostructures. Prussian blue nanoparticles (PBNPs) have received increasing attention in the electrochemical sensor field due to their enhanced surface to volume ratio and increased electrochemical properties associated with their use. The properties of nanoparticles are influenced by the type of synthesis and also by the type of chemical environment which characterizes them. For this reason, different types of PBNPs have been developed that can be produced alone [15] or in the presence of other nanomaterials in order to constitute a hybrid nanocomposite [16,17] each characterized by particular properties.

However, the fabrication of sensors can require several steps and each step introduces variability in the performance of the resulting sensors. This necessitates the development of fabrication procedures which are rapid and simple while also being highly reproducible. The use of printing techniques has been applied to the fabrication of electrochemical sensors for two decades now. In recent times, inkjet printing has become a powerful deposition technology for the fabrication of sensors. This deposition methodology has the capability to deliver precisely patterned quantities of ink at picolitre drop volumes and is now widely used as a tool for device fabrication [18–20]. The deposition of nanomaterial-based inks using inkjet printing has become a key technology for printed electronics [21].

The aim of this work was to develop an electrochemical sensor for the determination of H_2_O_2_ which would be simple and low cost to manufacture while also having excellent analytical performance, by depositing PBNPs onto screen-printed electrodes using inkjet printing. To the best of our knowledge, Hu *et al.* [22] reported an inkjet printing process to fabricate sensing devices for H_2_O_2_ based on PBNPs. However, this work reports important improvements in terms of sensitivity, detection limit, linear range and reproducibility regarding the determination of H_2_O_2_.

## 2. Experimental Section

### 2.1. Screen-Printed Electrode Fabrication

Screen-printed electrodes were produced with a 245 DEK (Poole, UK) screen-printing machine [23,24] using graphite-based conductive ink (Electrodag 421) for the working and counter electrode and Ag/AgCl conductive ink (Electrodag 477 SS) for the pseudo-reference electrode, obtained from Acheson (Milan, Italy). The insulating layer was Vinilflat 38.101E from Argon (Milan, Italy). The substrate was a flexible polyester film (Autostat HT5) obtained from Autotype (Milan, Italy). The diameter of the working electrode was 0.3 cm resulting in an apparent geometric area of 0.07 cm^2^.

### 2.2. Chemical Reagents

All chemicals from commercial sources were of analytical grade. All solutions were prepared using distilled water.

### 2.3. Preparation of PBNPs Dispersion

**Figure 1.**
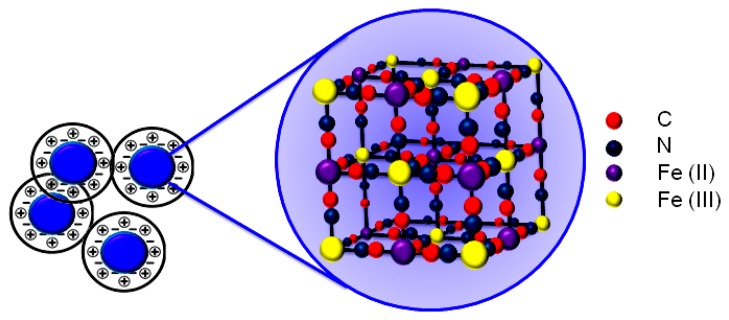
Schematic representation of Prussian blue nanoparticle lattice structure.

The PBNP dispersion was synthesized according to Chen *et al.* [25] by mixing equimolar amounts of potassium ferrocyanide (K_4_[Fe(CN)]_6_) and iron (III) chloride (FeCl_3_) in presence of HCl in acidic conditions. Briefly, 2 mM K_4_[Fe(CN)]_6_ (2 mL) was mixed with 0.1 M KCl (1 mL) in 10 mM HCl. Subsequently, 2 mM FeCl_3_ (2 mL) was added dropwise into the K_4_[Fe(CN)]_6_ solution under vigorous stirring. A blue solution was gradually formed and the reaction was allowed to proceed overnight to make the reaction complete. The obtained colloid solution was stable for three weeks. A schematic of the structure of the PB crystal is shown in Figure 1.

### 2.4. SPE Modification by Inkjet Printing

Inkjet printing was performed using a Dimatix DMP 2831 piezoelectric printer with the Dimatix Drop Manager software (FUJIFILM Dimatix Inc., Santa Clara, CA, USA) a drop spacing of 20 μm using all 16 nozzles. Inkjet-printed sensors were stored dry at room temperature up to 2 months without any loss of activity towards hydrogen peroxide detection.

### 2.5. Apparatus

Electrochemical measurements were carried out using a Bio-Logic SP-200 potentiostat equipped with EC-Lab^®^ software (Bio-Logic Science Instruments, Claix, France). UV-Vis measurements were obtained using a Lambda Bio spectrophotometer (Perkin Elmer, Waltham, MA, USA). Micrographs of the bare SPE and modified SPE with PBNPs were obtained by field emission scanning electron microscopy (FEG-SEM, Leo Supra 35, Oberkochen, Germany).

## 3. Results and Discussion

### 3.1. Physical and Electrochemical Characterization of Prussian Blue Nanoparticle-Modified Electrodes

**Figure 2.**
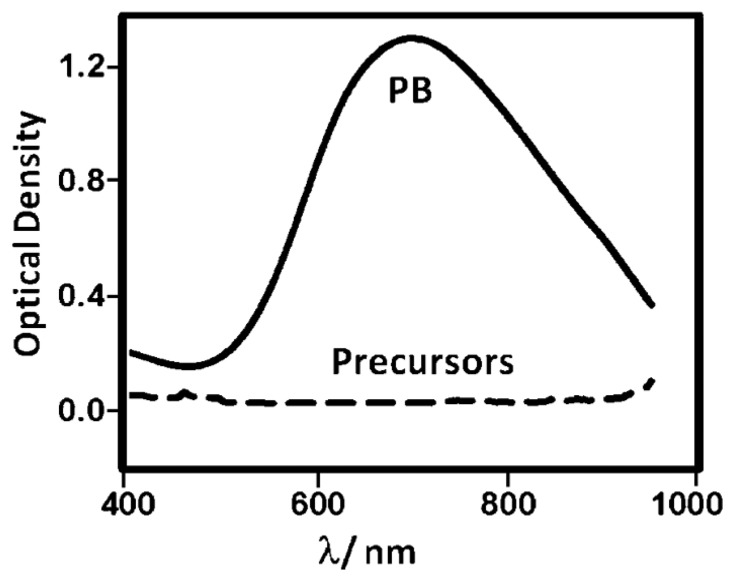
UV-vis spectra of the PBNP dispersion and its precursors.

**Figure 3.**
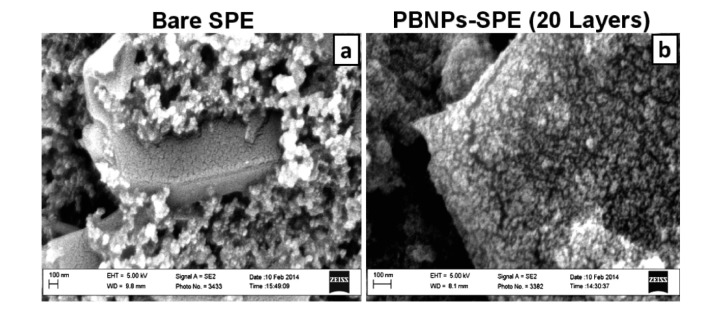
Scanning electron micrographs of (**a**) bare SPE and; (**b**) SPE modified with 20 inkjet-printed layers of PBNPs. Acceleration voltage: 5 keV, magnification 10 k.

The dispersion of PBNPs was characterized using UV-visible spectroscopy and scanning electron microscopy (SEM). The UV-visible spectra of the diluted PBNPs (Figure 2) revealed a broad band centered at 700 nm due to the Fe^II^ to Fe^III^ charge-transfer, characteristic of Prussian blue as seen in previous literature [26]. The synthetic precursor materials showed little distinctive absorption properties across this wavelength range. Screen-printed carbon electrodes (SPEs) were also analysed by SEM before and after deposition of the PBNP ink (Figure 3). Following deposition, the graphitic flakes were covered by a reasonably homogeneous layer of PBNPs with an average diameter of 15 nm and variation of approximately 12%.

**Figure 4.**
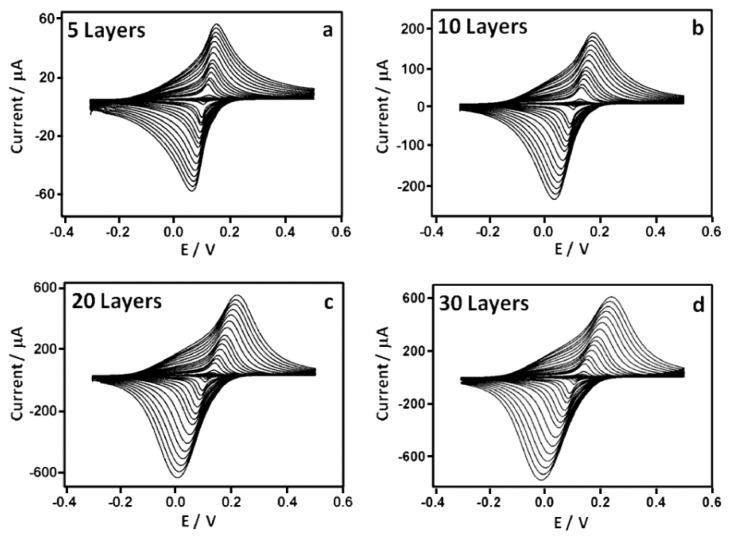
Cyclic voltammograms of PBNP-modified SPEs with (**a**) 5, (**b**) 10, (**c**) 20 and (**d**) 30 layers. Scan rates were from 10 to 1000 mV·s^−1^*vs*. Ag/AgCl in 0.05 M phosphate buffer, 0.1 M KCl, pH 7.4.

The effect of the quantity of PBNPs deposited onto the SPEs on the electrochemical properties of the sensors was investigated by performing different numbers of prints. Electrodes were modified with 5, 10, 20 and 30 layers of the PBNP solution. Cyclic voltammetric analysis of the sensors was performed in 0.05 M phosphate buffer containing 0.1 M KCl (pH 7.4) at scan rates between 10 and 1000 mV *vs*. Ag/AgCl in the potential range from −0.3 V to 0.5 V (Figure 4). A pair of redox peaks can be seen which are due to the oxidation and reduction of Prussian blue in which the PB is reduced to PW which is re-oxidized to PB. PB is known to exist in both a soluble and insoluble form, according to the following reactions, respectively:

(1) $${\text{KFe}}^{III}\left[{\text{Fe}}^{II}{\left(\text{CN}\right)}_{6}\right]+{\text{K}}^{+}+{\text{e}}^{-}⇆{\text{K}}_{2}{\text{Fe}}^{II}[{\text{Fe}}^{II}{\left(\text{CN}\right)}_{6}$$

and:

(2) $${{\text{Fe}}_{4}}^{III}{\left[{\text{Fe}}^{II}\left(\text{CN}\right)\right]}_{6}+4{\text{K}}^{+}+4{\text{e}}^{-}⇆{\text{K}}_{4}{{\text{Fe}}_{4}}^{II}{\left[{\text{Fe}}^{II}{\left(\text{CN}\right)}_{6}\right]}_{3}$$

The solubility of PB depends on the ease with which the potassium ion enters and leaves the film. In establishing the properties of the PB film, it is important to establish whether the soluble or insoluble form has been fabricated. There are several methods with which an evaluation of the electron transfer processes involved can be achieved. One method is to employ the peak width at half height (δ_0.5_), which tends towards 90.6/*n* mV when both the cathodic and anodic peaks tend to reversibility, where *n* is the number of electrons involved [27]. The δ_0.5_ was also calculated by taking into account the separation achieved at 10 mV·s^−1^ for the various electrodes, yielding a value of 21 ± 0.8 mV for all four sensors analysed. Peak separation at low scan rate was used to ensure operation in the reversible regime. In this case, *n* was found to be equal to 4.3 ± 0.3, which suggests the presence of the insoluble form of PB (Equation (2)). This reaction involves four electrons and four potassium ions which enter and leave the film in order to ensure charge neutrality of the crystal lattice during the redox process.

**Figure 5.**
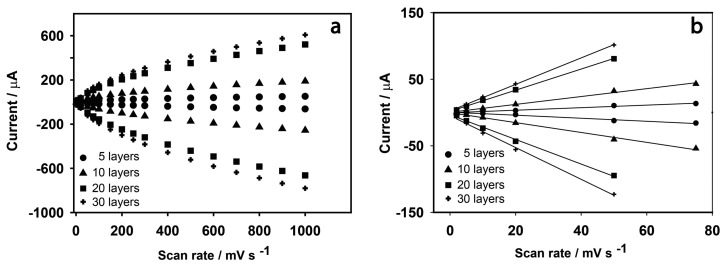
(**a**) Current *vs*. scan rate (10 to 1000 mV·s^−1^*vs*. Ag/AgCl) and; (**b**) linear correlation between current and scan rate from 0 to 80 mV·s^−1^ for PBNPs-SPEs, demonstrating thin layer behavior.

**Figure 6.**
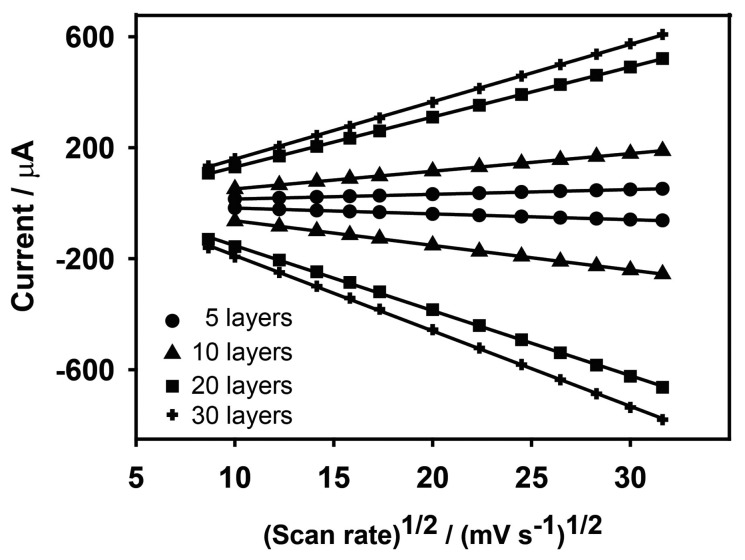
Current *vs*. square root of scan rate for PBNP-SPEs demonstrating the diffusion-controlled behavior.

Anodic and cathodic peak currents were also related to the number of deposited layers. *I*_p,a_ values were 1.11, 7.10, 18.9 and 22.1 μA and *I*_p,c_ values were 0.99, 5.901, 17.8 and 18.0 μA for 5, 10, 20 and 30 layers, respectively. Anodic and cathodic currents may vary linearly with scan rate (*v*), or by *v*^1/2^, depending on whether electron transfer processes are surface confined, or whether they depend on diffusion. Figure 5 shows the dependency of the peak current responses of the electrodes on scan rate across the entire scan rate range, and in detail between 0 and 80 mV·s^−1^, while Figure 6 shows the dependency of *I*_p_ to *v*^1/2^. This demonstrates that, for fewer layers of PBNPs (5 or 10 prints) at lower scan rates, currents increase linearly with *v* and suggest a surface controlled process [28], while for thicker films or at higher scan rates, peak currents were proportional to *v*^1/2^ due to the fact that the electrochemical process was controlled by diffusion of the counter ion, K^+^, in the lattice. Table 1 shows the anodic currents and slopes at 50 mV·s^−1^ for different numbers of deposited layers of PBNPs. This data suggests that, while peak current increases with layer thickness, little gain was achieved by moving from 20 to 30 layers, between which, there was also relatively little difference in their electron transfer rates. As a consequence, sensors fabricated with 20 layers were selected for further work.

### 3.2. Electrochemical Characterization of Hydrogen Peroxide at a PBNP–SPE

**Figure 7.**
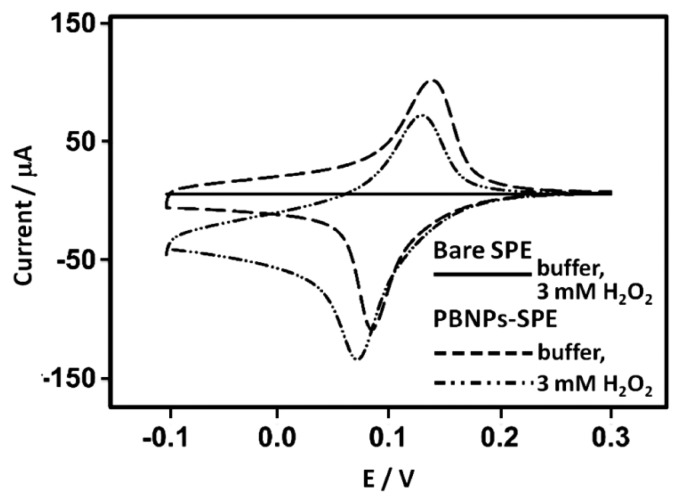
Cyclic voltammograms of bare SPE in 3 mM H_2_O_2_ (solid line), SPE modified with 20 layers of PBNP dispersion in buffer (dashed line) and 3 mM H_2_O_2_ (dashed-dotted line). All in 0.05 M phosphate buffer, 0.1 M KCl, pH 7.4 at 50 mV·s^−1^*vs*. Ag/AgCl.

The behavior of PBNP-SPEs toward hydrogen peroxide reduction was investigated using cyclic voltammetry in 0.05 M phosphate buffer containing 0.1 M KCl (pH 7.4) in a potential range from −0.1 V to 0.3 V (*vs*. Ag/AgCl) with a scan rate of 50 mV/s. Bare and PBNP-modified SPEs were studied in the presence and absence of H_2_O_2_ (Figure 7). At bare SPEs, there was little or no visible reduction of H_2_O_2_ at the electrode. For PBNP-SPEs, the characteristic redox couple for PB could be seen, while in the presence of 3 mM H_2_O_2_, the cathodic peak, indicating the formation of PW was shifted to a more negative potential of 14 mV, accompanied by an increase in the cathodic current. The anodic peak was also shifted to lower potentials and was reduced in peak current, as expected for mediated, reductive reactions.

**Figure 8.**
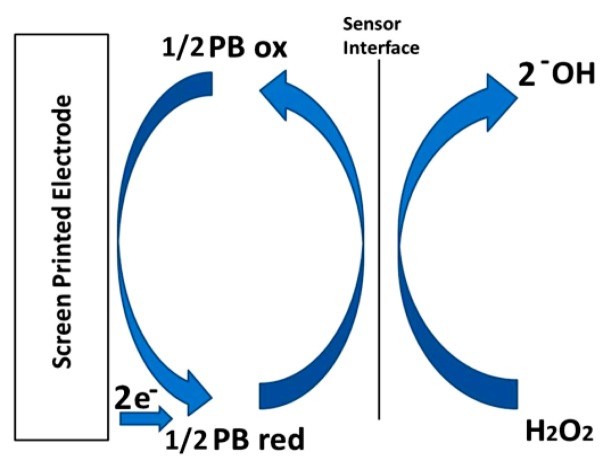
Mechanism of the catalytic H_2_O_2_ reduction mediated by PB.

These results demonstrate that the PBNP-modified SPEs have electrocatalytic activity towards hydrogen peroxide reduction according to the scheme in Figure 8 and Equations (3) and (4) [12] and related Equations (3) and (4).

(3) $${\text{K}}_{4}{{\text{Fe}}_{4}}^{II}{\left[{\text{Fe}}^{II}{\left(\text{CN}\right)}_{6}\right]}_{3}+2H_{2}{\text{O}}_{2}⇆{{\text{Fe}}_{4}}^{III}{\left[{\text{Fe}}^{II}\left(\text{CN}\right)\right]}_{6}+4{\text{OH}}^{-}+4{\text{K}}^{+}$$

(4) $${\text{H}}_{2}{\text{O}}_{2}+2{\text{e}}^{-}\to 2{\text{OH}}^{-}$$

Further work was performed to measure the quantitative response of the sensors to H_2_O_2_.

### 3.3. Amperometric Detection of Hydrogen Peroxide

**Figure 9.**
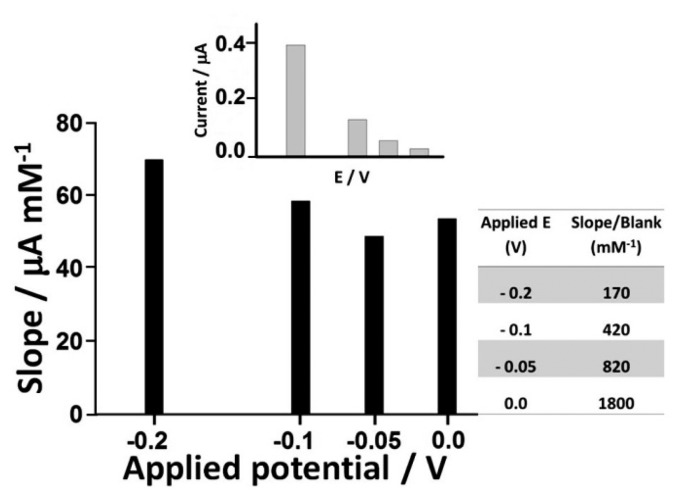
Selection of the optimum applied potential for the amperometric measurement of H_2_O_2_.

The choice of the optimum applied potential at the working electrode is essential in achieving the lowest possible limit of detection. Several factors are relevant when making this selection. One is the signal to background ratio of the sensor at a particular potential and the non-faradaic processes that result from a potential step which contributes to the background signal and reduces achievable detection limits. However, this must be balanced against the greater levels of electrolysis that can be achieved at more extreme potentials. The other factor is the likely increase in electrochemical interference at more extreme potentials. The slope to blank ratios of the sensors at 0, −0.05, −0.1 and −0.2 V (*vs*. Ag/AgCl) are shown in Figure 9. The sensors showed the greatest slope to blank ratio at 0 V, with a response ratio some ten-fold higher than at −0.2 V. This, coupled to the significant reduction in interference from a number of important reductants at this potential, as reported in a number of earlier studies resulted in a preference for operation of the sensor at 0 V *vs*. Ag/AgCl [29–31].

**Figure 10.**
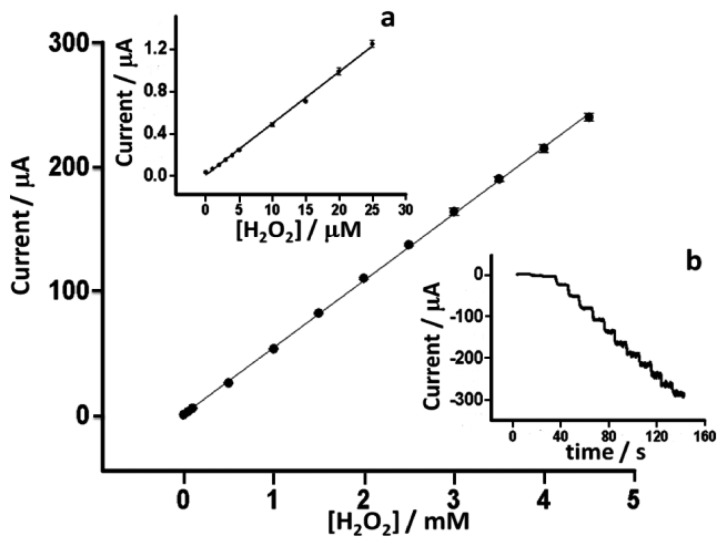
Calibration curve of H_2_O_2_ on PBNP-SPEs (n = 3) in 0.05 M phosphate buffer, 0.1 M KCl, pH 7.4 at 0 V *vs*. Ag/AgCl. (**a**) Detail from 0 to 25 μM and (**b**) Raw amperometric data from 0 to 4.5 mM.

Detailed characterization of the response of the PBNP-SPEs towards H_2_O_2_ was performed at 0 V *vs*. Ag/AgCl pseudoreference on electrodes modified with 20 layers of inkjet-printed PBNPs. Measurements were performed in 0.05 M phosphate buffer containing 0.1 M KCl (pH 7.4) in a stirred batch reaction vessel (n = 6). Limit of detection, linear range, sensitivity and reproducibility were studied (Figure 10). Sensors showed excellent linearity between 0 and 4.5 mM with a detection limit of 0.2 μM, obtained from the formula LOD = 3·σ_b_/slope (r^2^ = 0.9994). The sensitivity was 762 μA·mM^−1^·cm^−2^. Furthermore, the sensors showed excellent inter-electrode relative standard deviation of less than 5% for all concentrations tested. As well as the excellent analytical features demonstrated by these electrodes, this is also coupled to the ease and simplicity of their fabrication and amenability to mass production. This compared favorably with several earlier works (Table 2). Hu *et al.* [22] achieved an LOD of 20 μM and a sensitivity of 164.82 μA·M^−1^·cm^−2^. Haghighi *et al.* [15] achieved an analytical range from 2.1 μM to 0.14 mM by using adsorbed PBNPs covered with a layer of Nafion reaching a sensitivity of 138.6 μA·mM^−1^·cm^−2^, but which was more than 5-fold less sensitive than the current sensor, also with a 5-fold higher LOD. Yang *et al*. [32] reported a PB-based electrochemical sensor based on chitosan functionalized with graphene nanosheets, and obtained a similar level of sensitivity to the PBNP inkjet-printed device (816.4 μA·mM^−1^·cm^−2^) but with a higher LOD and narrow linear range (10 μM to 0.4 mM). In other work, Karyakin *et al*. [13] achieved excellent detection limits. However, this was achieved using a gold ultramicroelectrode with electrochemical modification, which requires electrochemical cleaning and deposition processes and so makes mass fabrication challenging. The approach followed by Cao *et al*. [17] also achieved good performance in terms of limit of detection but was also characterized by a long and complex synthesis of the electrocatalyst, while also having a relatively narrow dynamic range. In comparison to other procedures, the inkjet-printed PBNP-modified electrodes certainly represent a good balance between excellent analytical performance and low cost reproducible mass production.

## 4. Conclusion

The fabrication of sensors for the measurement of H_2_O_2_ was achieved using screen-printed electrodes modified with inkjet-printed Prussian blue nanoparticles. Sensors fabricated with 20 inkjet-printed layers were shown to have excellent limits of detection, linear range and reproducibility. The developed sensor represents a simple, robust and reliable means of sensing H_2_O_2_ which avoids long and complex multi-step sensor fabrication processes and is suitable for large scale mass production and has many potential applications in environmental, industrial and physiological monitoring, particularly where single use and disposability are advantageous.

## Figures and Tables

**Table 1.** Summary of electrochemical parameters from scan rate studies (50 mV·s^−1^*vs*. Ag/AgCl) for electrodes modified with different numbers of inkjet-printed layers of PBNPs in 0.05 M phosphate buffer, 0.1 M KCl, pH 7.4.

PBNPs Layers	Anodic Current @ 50 mV·s^−1^	Slope (Thin Layer) μA·mV^−1^·s	Slope (Diffusion Controlled) μA·mV^−1/2^·s^1/2^
● 5	10.3	0.152	1.69
▴ 10	32.4	0.628	6.37
▪ 20	80.8	1.83	17.9
+ 30	101	2.32	20.7

**Table 2.** Analytical performance characteristics of a number of Prussian blue-modified electrodes for H_2_O_2_ detection.

Electrode	Modification Procedure	Sensitivity (μA·mM^−1^·cm^−2^)	LOD (μM)	Linear Range (mM)	Reference
PB-GU E	Electrodeposition	2200	0.01	10^−5^–1	[13]
Nafion-PBNPs-GE	Dipping	138	1	0.002–0.14	[15]
PBNCs/rGO-GCE	Drop Casting	*	0.045	5 × 10^−5^–0.12	[17]
PBNPs (100 nm)-SPE	Inkjet	0.164	20	0.02–0.7	[22]
PBNPs (15 nm)-SPE	Inkjet	762	0.2	0.001–4.5	This work

PB-GUμE: Prussian Blue gold ultramicroelectrode (125 μm); GE: Graphite Electrode; PBNCs/rGO-GCE: Prussian Blue NanoCubes on the surface of reducede Graphene Oxide adsorbed on Glassy Carbon Electrode;

* Area of the working electrode has not been reported in the cited paper.
